# The relationship between poison frog chemical defenses and age, body size, and sex

**DOI:** 10.1186/s12983-015-0120-2

**Published:** 2015-10-01

**Authors:** Adriana M. Jeckel, Ralph A. Saporito, Taran Grant

**Affiliations:** Department of Zoology, Institute of Biosciences, University of São Paulo, 05508-090 São Paulo, São Paulo Brazil; Department of Biology, John Carroll University, University Heights, OH 44118 USA

**Keywords:** Alkaloids, Amphibia, Anura, Bufonidae, Bufotenine, *Melanophryniscus*, Sequestration, Skeletochronology

## Abstract

**Introduction:**

Amphibians secrete a wide diversity of chemicals from skin glands as defense against predators, parasites, and pathogens. Most defensive chemicals are produced endogenously through biosynthesis, but poison frogs sequester lipophilic alkaloids from dietary arthropods. Alkaloid composition varies greatly, even among conspecific individuals collected at the same time and place, with some individuals having only a few micrograms of one or a few alkaloids and others possessing >1 mg of >30 alkaloids. The paucity of alkaloids in juveniles and their abundance in adults suggests that alkaloids accumulate over time; however, alkaloid diversity is highly variable among adult poison frogs and has never been studied in relation to individual age. Using skeletochronology to infer individual ages and gas chromatography–mass spectrometry and vapor phase Fourier-transform infrared spectral analysis to identify the defensive chemicals of 63 individuals, we tested the relationship between defensive chemicals and age, size, and sex in the Brazilian red-belly toad, *Melanophryniscus moreirae*, a poison frog that possesses both sequestered alkaloids and the biosynthesized indolealkylamine bufotenine.

**Results:**

Adult females were, on average, older and larger than adult males. Juveniles were smaller but not necessarily younger than adults and possessed bufotenine and 18 of the 37 alkaloids found in adults. Alkaloid richness was positively related to age, but not size, whereas the quantities of sequestered alkaloids and bufotenine were positively related to size, but not age. Defensive chemicals were unrelated to sex, independent of size.

**Conclusions:**

The relationship between alkaloid richness and age appears to result from the gradual accumulation of alkaloids over a frog’s lifetime, whereas the relationship between the quantity of defensive chemicals and size appears to be due to the greater storage capacity of larger individuals. The decoupling of age and size effects increases the amount of individual variation that can occur within a population, thereby possibly enhancing anti-predator efficacy. Further, given that both richness and quantity contribute to the overall chemical defense of individual frogs, our results suggest that older, larger individuals are better defended than younger, smaller ones. These considerations underscore the importance of including age in studies of the causes and consequences of variation in poison frog chemical defenses.

**Electronic supplementary material:**

The online version of this article (doi:10.1186/s12983-015-0120-2) contains supplementary material, which is available to authorized users.

## Introduction

The use of chemicals as defensive agents is widespread among plants, animals, and micro-organisms. In addition to defensive chemicals that are biosynthesized endogenously, many organisms sequester defensive chemicals from environmental sources. This phenomenon has been best studied in insects [1], but it also occurs in marine invertebrates [2, 3] and terrestrial vertebrates [4]. One of the most salient characteristics of systems of chemical defense is the extreme variation in defensive chemicals often observed even among individuals of the same population, and a central goal of studies of chemical defense is to understand the causes and consequences of this variation [5].

Amphibians secrete a wide diversity of chemicals from granular glands in the skin to defend themselves against predators and pathogens (e.g. [6–10]). Most defensive chemicals are produced endogenously through biosynthesis, but poison frogs obtain lipophilic alkaloids through sequestration from dietary arthropods [11–13]. To date, over 1200 sequestered alkaloids representing 28 structural classes have been detected in poison frogs ([14] and papers cited therein). Studies of individual frogs have revealed that the richness and quantity of sequestered alkaloids vary greatly within species, not only among samples taken at different times and places, but also among individuals collected at the same time and place, with some individuals having only a few micrograms of one or a few alkaloids and others possessing more than 1 mg of more than 30 alkaloids (e.g. [15–24]). Similarly, although sequestered alkaloids generally are considered the main defensive chemicals in poison frogs, some species also possess variable quantities of endogenously biosynthesized defensive chemicals, such as the indolealkylamine bufotenine [14].

The causes of variation in poison frog chemical defenses are poorly understood. Adult female amphibians tend to be larger [25], older [26], and often have more alkaloid defenses [22] than males. Given that alkaloid richness and quantity are often greater in larger individuals (e.g. [22]), sexual size dimorphism could explain why females possess more alkaloids than males; however, adult females of the strawberry poison frog, *Oophaga pumilio*, are the same size as males yet possess greater alkaloid richness and quantity [20]. The paucity of alkaloids in juveniles and their abundance in adults [27–29] suggests that alkaloids accumulate over time; however, chemical defenses are highly variable among adult poison frogs, and, although alkaloid diversity has been studied in relation to body size and sex, it has never been examined in relation to individual age.

In this study, we tested the relationship between defensive chemicals (sequestered alkaloids and biosynthesized bufotenine) and age, body size, and sex in the Brazilian red-belly toad, *Melanophryniscus moreirae*, a poison frog from the Serra da Mantiqueira plateau in southeastern Brazil.

## Results

### Age and size

Adult females and males were 3–6 and 2–6 years old, respectively (Table 1). Snout–vent length (SVL) was sexually dimorphic in adults (sexual dimorphism index = 0.13), with females being significantly longer (*t*_53_ = −9.5635, *P* < 0.0001) and older (*t*_53_ = −2.5116, *P* = 0.0151) than adult males, suggesting that sexual size dimorphism might be due to the different age structure of each sex [26]; however, the mean SVL of same-aged adult females and males differed significantly at all ages (Additional file 1), which shows that age structure merely exacerbates an effect that is already present within age cohorts.

**Table 1:** *Melanophryniscus moreirae* age structure

Sex	*n*	Mean size ± SE (mm)	Mean age ± SE (years)	Median age (years)	Modal age (years)	AM (years)	Mean size at AM ± SE (mm)	Longevity (years)	PRLS (years)
Adult male	40	23.2 ± 0.2	4.2 ± 0.2	4	4	2	21.7 ± 0.9	6	4
Adult female	15	26.2 ± 0.2	4.9 ± 0.3	5	5 and 6	3	26.3 ± 0.0	6	3
Juvenile female	7	16 ± 1.3	2.0 ± 0.5	–	–	–	–	–	–

*AM* age at maturity, age of the youngest adult, *PRLS* potential reproductive lifespan; Size: snout–vent length

The seven juveniles were females 1–5 years old. Despite the overlap in age between adult and juvenile females, there was no overlap in SVL, suggesting that female sexual maturity is determined by size, not age. The oldest juvenile was 22.2 mm SVL, which was considerably smaller than both the mean age of maturity (Table 1) and the smallest adult female, a 6-year-old (one of the oldest females) of 24.8 mm SVL.

### Juvenile chemical defenses

Juvenile skins weighed 32.4–281.4 mg (87.5 ± 93.9 mg) and contained 18 alkaloids of seven structural classes (Additional file 2). Bufotenine occurred in only three of the seven juveniles. Alkaloid richness (1–12 alkaloids; 4.9 ± 1.6 alkaloids), alkaloid quantity (1.5–331.1 μg; 108.4 ± 50.4 μg), and bufotenine quantity (0.0–121.3 μg; 22.4 ± 16.9 μg) were highly variable. Only allopumiliotoxin (aPTX) **323B** was detected in all juveniles, followed by aPTX **337D** in four juveniles, pumiliotoxin (PTX) **267C** and PTX **265D** in three, and all remaining alkaloids (5,8-I, 5,6,8-I, Tricyclic, hPTX, and unclassified) in only one or two juveniles. All defensive chemicals found in juveniles were also found in adults, which possessed 37 alkaloids and bufotenine [14].

### Chemical defenses, age, size, and sex

**Fig. 1:**
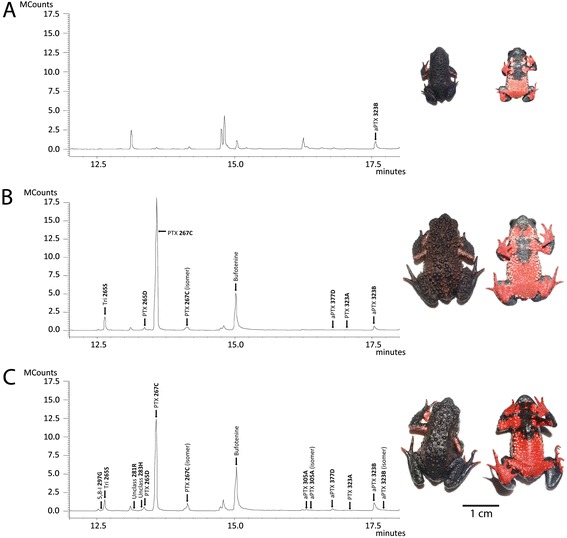
Gas chromatograms and dorsal and ventral images of three individuals of *Melanophryniscus moreirae* of different ages and sizes, which are representative of the 63 individuals included in this study. Unidentified peaks are non-alkaloids (e.g. fatty acid methyl esters) that remained following fractionation. **a** 1-year-old juvenile female: 36.8 mg skin mass, 62.1 μg defensive chemicals, one alkaloid, no bufotenine (MZUSP 154148). **b** 3-year-old adult male: 239.2 mg skin mass, 470.0 μg defensive chemicals, seven alkaloids, bufotenine present (MZUSP 154119). **c** 6-year-old adult male: 194.6 mg skin mass, 305.1 μg defensive chemicals, 13 alkaloids, bufotenine present (MZUSP 154112)

The non-parametric multiple regression analyses showed that alkaloid richness was related to individual age, but not skin mass, whereas the quantities of both sequestered alkaloids and bufotenine were related to skin mass, but not age (Table 2; Fig. 1). The response variables were not significantly related to sex. These findings were insensitive to the method of age calculation used to infer individual ages (Additional file 3), as were the relationships between age and alkaloid richness and skin mass and bufotenine quantity following removal of juveniles from the analyses (Additional file 4).

**Table 2:** Non-parametric multiple regression analyses of Brazilian red-belly toad chemical defenses in relation to sex, skin mass, and age

	Alkaloid richness			Alkaloid quantity			Bufotenine quantity		
	Sex	Skin mass	Age	Sex	Skin mass	Age	Sex	Skin mass	Age
Regression coefficient	0.9548	0.0088	1.2622	112.156	1.428	48.769	13.6241	0.6271	1.5966
*P*	0.3093	0.3090	0.0058*	0.0638	0.0079*	0.0755	0.4703	0.0003*	0.8564
*R* ^*2*^	0.2990			0.3750			0.3387		
*R* ^*2*^-adj	0.2628			0.3426			0.3045		
*F*	8.2490			11.6004			9.9057		
*P*	0.0001*			0.0001*			0.0001*		

Regression coefficients, *R* ^2^, adjusted *R* ^2^ (*R* ^2^-adj), and *F*-statistics for the full models (3 and 58 degrees of freedom), and respective *P*-values from non-parametric multiple regression analyses (9999 permutations; two-tailed tests)

*Statistically significant *P*-values

## Discussion

Our finding that all of the sampled juveniles were females was unexpected; however, given our limited sample (*n* = 7), we cannot rule out the possibility that this sexual bias is simply an artifact due to small sample size. Collecting data on juveniles following metamorphosis and initial emigration from breeding sites is notoriously challenging in anurans generally and *Melanophryniscus* specifically. For example, in a study of migratory movement in *M. cambaraensis*, no juveniles were detected over 5 months of sampling using drift fences with pit fall and funnel traps as well as visual searches [30, 31]. Similarly, among the 48 specimens of *M. macrogranulosus* collected from 1960 to 2013, only six were juveniles [32].

In organisms that sequester defensive chemicals, age-related differences in chemicals are often mediated by shifts between life stages that result in different diets. For example, the larvae of many lepidopterans sequester plant secondary metabolites that can either be lost or retained at metamorphosis [33]. Poison frogs sequester alkaloids from terrestrial arthropods [11, 12], which explains the paucity of alkaloids in late-stage aquatic tadpoles and recently metamorphed individuals of species that lack maternal provisioning [29]. Similarly, the diets of adult and juvenile poison frogs differ [34], which could explain differences in defensive chemicals between adults and juveniles; however, dietary differences between juveniles and adults cannot account for differences in alkaloid richness among adults and, therefore, cannot be responsible for the significant positive relationship between age and alkaloid richness among adults when juveniles are excluded from analyses. Instead, we suggest that this relationship owes to the gradual accumulation of sequestered alkaloids over a frog’s lifetime. Wild-caught frogs retain large amounts of alkaloids for at least 6 years after capture [15, 27, 35, 36], and the variation in alkaloid profiles observed in poison frog samples collected at the same localities over multiple years [18, 21] indicates extensive temporal variation in either the local availability of alkaloid-containing arthropods or the alkaloids these arthropods contain. Consequently, it follows that, on average, longer-lived individuals will have sequestered a greater diversity of alkaloids, regardless of their growth rate and size.

We also found that the quantities of both sequestered alkaloids and bufotenine are positively related to body size. Although it could be hypothesized that larger individuals consume greater quantities of arthropods and, therefore, greater quantities of alkaloids, given that bufotenine is biosynthesized, not sequestered, it is unlikely that this relationship is due to diet. Instead, we hypothesize that the increase in the quantity of defensive chemicals with body size is due to increased storage capacity in larger individuals. In *Oophaga pumilio* there is a positive allometric relationship of granular gland size and number with body size, which allows larger individuals to store more alkaloids [37]. Although information on gland size and number is unavailable for red-belly toads, increased storage capacity would account for both the greater quantities of sequestered alkaloids and biosynthesized bufotenine in larger individuals and also the surprisingly low quantities of bufotenine observed in juveniles. Even though bufotenine was the second most abundant chemical in adults, it was only present in the three juveniles that also possessed the greatest quantity of alkaloids.

We did not find a significant relationship between sex and either the richness or quantity of defensive chemicals in *Melanophryniscus moreirae*. This result is at odds with previous findings in *Oophaga pumilio* in which females possessed significantly greater richness and quantity of sequestered alkaloids than males [20]. Saporito et al. [20] did not include individual age in their analysis, but they hypothesized that the differences between males and females (which did not differ in size) were due to either sexually dimorphic behaviors that resulted in differences in consumption of alkaloid-containing arthropods or different predation pressures for each sex. Based on our results, we suggest that those factors are likely to be related to sexual differences in alkaloid quantity, but that differences in alkaloid richness are most likely due to different age structures, with females being, on average, older than males.

In addition to elucidating the causes of variation in poison frog chemical defenses, the relationship between defensive chemicals, age, and size is also relevant for understanding the consequences of this variation. For example, avian predators find variation in prey chemical defenses to be aversive, presumably because they avoid uncertainty when managing intake of chemicals [38]. The decoupling of variation in chemical defenses in relation to age and size in poison frogs increases the amount of individual variation that can occur within a population, thereby possibly enhancing anti-predator efficacy. Similarly, inhibition of microbial growth appears to depend on both the quantity of alkaloids and the richness of alkaloid cocktails [39], suggesting that older and larger individuals might be better defended against a broader diversity of pathogens than younger and smaller individuals.

## Conclusions

We demonstrate for the first time that the chemical defenses of post-metamorphic poison frogs vary in relation to age. The positive relationship between alkaloid richness and age appears to result from the gradual accumulation of sequestered alkaloids over a poison frog’s lifetime. We also found that the quantities of sequestered alkaloids and synthesized bufotenine are positively related to the size of each individual, which appears to be due to the greater storage capacity of larger individuals. The decoupling of age and size effects increases the amount of individual variation that can occur within a population, which might enhance anti-predator efficacy. Further, given that both richness and quantity contribute to chemical defense, our results suggest that older, larger individuals are better defended than younger, smaller ones. These considerations underscore the importance of including age as an explanatory variable to understand the causes and consequences of variation in poison frog chemical defenses.

## Methods

### Specimens

A total of 63 *Melanophryniscus moreirae* (41 males, 15 females, 7 juveniles) were collected in Itatiaia National Park (Serra da Mantiqueira, Rio de Janeiro, Brazil, GPS coordinates: 22°23′05.88″S, 44°40′41.83″W, WGS84 datum) on 30 November 2013. Skins were removed completely using 3 mm Vannas spring scissors, fine forceps, and a Zeiss Stereo Discovery V8 microscope, weighed (to 0.1 mg), and stored in 100 % methanol in individual 4 mL glass vials with Teflon-coated lids prior to chemical analysis. Skinned specimens were fixed in 10 % formalin, preserved in 70 % ethanol, and deposited in the amphibian collection of the Museu de Zoologia da Universidade de São Paulo (MZUSP 154089-154151). Snout–vent length (SVL) was measured to 0.1 mm and sex was determined by examination of gonads. Males with vocal slits and nuptial pads were scored as adults and those lacking these structures were scored as juveniles. Females with enlarged, differentiated ova and convoluted oviducts were scored as adults and those with undifferentiated ova and narrow, straight oviducts were scored as juveniles.

### Age calculation

Individual differences in growth rate prior to first breeding can result in a variety of sizes within a given age class [40, 41], making body size an unreliable indicator of age in amphibians (e.g. [40, 42–44]). The most widely used method for determining the age of individual amphibians is skeletochronology [45], which infers age by counting incremental skeletal growth marks that are correlated with predictable, periodic events. In bone formed during periods of high metabolic activity, osteocytes deposit matrix quickly and blood vessels are produced. During periods of reduced metabolic rates, such as hibernation or estivation, growth slows and bones produce dense layers of osteocyte matrix that are organized into discrete, narrow lines of arrested growth (LAGs; [46]). The alternating pattern of thick rings of loose matrix followed by LAGs can be used to infer individual age. *Melanophryniscus moreirae* is endemic to the high altitude (1800–2400 m) mountain range Serra da Mantiqueira [47–49] where winter temperatures reach as low as −7 °C and dormant toads lie concealed in hibernacula 5–15 cm deep in soil and ravines [50], resulting in well-defined LAGs.

To visualize LAGs, bones were decalcified in a 15 % formic acid solution for 24–96 h, depending on size, embedded in paraffin wax, cross-sectioned at 7 μm using a rotary microtome, and stained with Mayer’s haematoxilin and eosin (modified from [51]). For each individual, we selected three sections from the middle portion of the diaphysis. Because skeletochronology has not been used previously in *Melanophryniscus*, we carried out a preliminary histological study of mid-diaphyseal sections of femora, humeri, and phalanges from 12 specimens and found that femora were most suitable for skeletochronological studies in *M. moreirae* (data not shown). Sections were analyzed and photographed with a Nikon Eclipse 80i light microscope equipped with a Nikon DS-Ri1 digital camera, and measurements were taken from images using ImageJ 1.48v [52]. For each individual, we measured the perimeter of the medullary cavity, resorption line, all visible LAGs, and the periosteal outer margin. We did not treat the periosteal outer margin as a LAG because specimens were collected approximately 2 months after emergence from hibernation and we had no way of determining if the outer growth ring was from the present or previous year. We were unable to reliably identify any LAGs in one specimen (adult male MZUSP 154131), which was excluded from analyses.

Bone remodeling can lead to age underestimation due to endosteal resorption and concomitant destruction of LAGs, requiring osteometric analysis to infer the occurrence and extent of resorption in each individual and, if necessary, apply a back calculation [46, 53, 54]. We estimated the number of missing LAGs by calculating the difference between the perimeters of the resorption line and the mean perimeters of each of the two visible LAGs of the five smallest individuals (13.2–15.0 mm SVL). If the difference exceeded 1 SD, we inferred that endosteal resorption resulted in LAG destruction in that individual; if the difference did not exceed 1 SD, we concluded that LAG destruction did not occur. Individual age was then inferred by summing the total number of observed and inferred LAGs. For each sex we calculated age at maturity (age of the youngest adult), mean SVL at age of maturity, longevity (oldest individual), potential reproductive lifespan (longevity-age at maturity), and mean, median, and modal age. We also used the methods of Sagor et al. [55], Guarino et al. [56], and Piantoni et al. [57] to assess the sensitivity of our results to the method of back calculation (see Additional file 3).

### Chemical analysis

Chemical data, skin mass, and sex were obtained previously for adults [14], and juvenile skins were analyzed following the same procedures and using the same instrumentation. Briefly, alkaloids and bufotenine were isolated from individual methanol extracts using an acid–base extraction [19]. Ten micrograms of nicotine ((−)-nicotine ≥99 %, Sigma-Aldrich) in a methanol solution (internal standard) and 50 μL of 1 N HCl were added to 1 mL of the original methanol extract. This combined methanol extract was concentrated with nitrogen gas to 100 μL and diluted with 200 μL of deionized water. This solution was then extracted four times, each time with 300 μL of hexane. The aqueous layer was then treated with saturated NaHCO_3_, followed by extraction three times, each time with 300 μL of ethyl acetate. The combined ethyl acetate fractions were dried with anhydrous Na_2_SO_4_, evaporated to dryness, and then reconstituted with methanol to 100 μL.

Gas chromatography–mass spectrometry (GC-MS) analysis was performed using a Varian Saturn 2100 T ion trap MS instrument coupled to a Varian 3900 GC with a 30 m 0.25 mm i.d. Varian Factor Four VF-5 ms fused silica column. GC separation was achieved by using a temperature program from 100 to 280 °C at a rate of 10 °C/min with helium as the carrier gas (1 mL/min). Alkaloid/bufotenine fractions were analyzed with both electron impact MS (EI-MS) and chemical ionization MS (CI-MS) with methanol as the CI reagent. Vapor phase Fourier-transform infrared spectral data (GC-FTIR) were obtained using a Hewlett-Packard model 5890 gas chromatograph, with an Agilent J&W DB-5 capillary column (30 m, 0.25 mm i.d., 0.25 μm), using the same temperature program as above, coupled with a Hewlett-Packard (HP) model 5971 Mass Selective Detector and a Hewlett-Packard Model 5965B IRD narrow range (4000–750 cm^−1^) infrared detector.

Individual alkaloids were identified by comparison of their observed MS properties (and FTIR properties for bufotenine) and GC retention times with those of previously reported anuran alkaloids (e.g. [14, 23, 24, 58]). Identification of bufotenine was based on comparison to reference standard: bufotenine solution, B-022, Cerilliant, Sigma-Aldrich. Isomers of known alkaloids were tentatively identified based on comparisons of EI mass spectral data and GC retention times. Individual frog skin extracts were analyzed in triplicate and the average quantity of defensive chemicals was determined by comparing the observed peak areas to the peak area of the nicotine internal standard, using a Varian MS Workstation v.6.9 SPI.

### Statistical analysis

All data are presented as mean ± SE. A significance level of 0.05 was applied in all statistical tests, with the sequential Bonferroni test applied when necessary [59]. We used *t*-tests to test for sexual dimorphism.

To test the relationship between the diversity of chemical defenses and individual age, body size, and sex, we performed multiple regression analyses using alkaloid richness (number of individual alkaloids), total alkaloid quantity (μg), and total bufotenine quantity (μg) per individual skin as response variables and age (years), skin mass (mg), and sex as explanatory variables. Because all juveniles in our sample were females, we pooled both sexes in our analyses. We used wet skin mass as a measure of body size instead of the more commonly used SVL because SVL is a one-dimensional measurement and, therefore, underestimates differences in body size; in contrast, because the skin envelops the entire body, skin mass is a more accurate measurement of body size. As some of the variables were not distributed normally (Shapiro-Wilk test), we used package ape 3.2 [60] in R Project 3.2.1 [61] to estimate significance (two-tailed tests) from 9999 random permutations (see Additional files 5 and 6).

## Additional files

Additional file 1:
**Results of** ***t*** **-tests comparing the sizes** **(snout–vent length)** **of adult females and males of the same age.** (PDF 34 kb) Additional file 2:
**Defensive chemicals ranked by quantity in individual skins of** ***Melanophryniscus moreirae*** **juveniles.** (PDF 69 kb) Additional file 3:
**Additional details on age calculation methods.** (PDF 140 kb) Additional file 4:
**Results of non-parametric multiple regression analyses of Brazilian red-belly toad chemical defenses in relation to sex, skin mass, and age following removal of juveniles.** (PDF 51 kb) Additional file 5:
**Data table including voucher numbers, sex, maturity, skin mass, alkaloid richness, alkaloid quantity, bufotenine quantity, age, and snout–vent length.** (TXT 3 kb) Additional file 6:
**Commands used to read the data table and perform non-parametric multiple regression analyses**. (TXT 2 kb)

## References

[1] Opitz SEW, Müller C (2009). Plant chemistry and insect sequestration. Chemoecology.

[2] Hay ME (1996). Marine chemical ecology: what’s known and what’s next?. J Exp Mar Bio Ecol.

[3] Paul VJ, Arthur KE, Ritson-Williams R, Ross C, Sharp K (2007). Chemical defenses: from compounds to communities. Biol Bull.

[4] Savitzky AH, Mori A, Hutchinson DA, Saporito RA, Burghardt GM, Lillywhite HB (2012). Sequestered defensive toxins in tetrapod vertebrates: principles, patterns, and prospects for future studies. Chemoecology.

[5] Speed MP, Ruxton GD, Mappes J, Sherratt TN (2012). Why are defensive toxins so variable? An evolutionary perspective. Biol Rev.

[6] Fritz G, Rand AS, Claude W (1981). The aposematically colored frog, *Dendrobates pumilio*, is distasteful to the large, predatory ant, *Paraponera clavata*. Biotropica.

[7] Macfoy C, Danosus D, Sandit R, Jones TH, Garraffo HM, Spande TF (2005). Alkaloids of anuran skin: antimicrobial function?. Z Naturforsch C.

[8] Rivas L, Luque-Ortega JR, Andreu D. Amphibian antimicrobial peptides and Protozoa: lessons from parasites. Biochem Biophys Acta. 1788;2009:1570–81. 10.1016/j.bbamem.2008.11.002 19046939

[9] Conlon JM (2011). The contribution of skin antimicrobial peptides to the system of innate immunity in anurans. Cell Tissue Res.

[10] Stynoski JL, Shelton G, Stynoski P (2014). Maternally derived chemical defences are an effective deterrent against some predators of poison frog tadpoles (*Oophaga pumilio*). Biol Lett.

[11] Saporito RA, Spande TF, Garraffo HM, Donnelly MA (2009). Arthropod alkaloids in poison frogs: a review of the “dietary hypothesis.”. Heterocycles.

[12] Saporito RA, Donnelly MA, Spande TF, Garraffo HM (2012). A review of chemical ecology in poison frogs. Chemoecology.

[13] Hantak MM, Grant T, Reinsch S, McGinnity D, Loring M, Toyooka N (2013). Dietary alkaloid sequestration in a poison frog: an experimental test of alkaloid uptake in *Melanophryniscus stelzneri* (Bufonidae). J Chem Ecol.

[14] Jeckel AM, Grant T, Saporito RA (2015). Sequestered and synthesized chemical defenses in the poison frog *Melanophryniscus moreirae*. J Chem Ecol.

[15] Daly JW, Secunda SI, Garraffo HM, Spande TF, Wisnieski A, Nishihira C (1992). Variability in alkaloid profiles in neotropical poison frogs (Dendrobatidae): genetic versus environmental determinants. Toxicon.

[16] Myers CW, Daly JW, Garraffo HM, Wisnieski A, Cover JF (1995). Discovery of the Costa Rican poison frog *Dendrobates granuliferus* in sympatry with *Dendrobates pumilio*, and comments on taxonomic use of skin alkaloids. Am Museum Novit.

[17] Mebs D, Pogoda W, Maneyro R, Kwet A (2005). Studies on the poisonous skin secretion of individual red bellied toads, *Melanophryniscus montevidensis* (Anura, Bufonidae), from Uruguay. Toxicon.

[18] Saporito RA, Donnelly MA, Jain P, Garraffo HM, Spande TF, Daly JW (2007). Spatial and temporal patterns of alkaloid variation in the poison frog *Oophaga pumilio* in Costa Rica and Panama over 30 years. Toxicon.

[19] Saporito RA, Donnelly MA, Garraffo HM, Spande TF, Daly JW (2006). Geographic and seasonal variation in alkaloid-based chemical defenses of *Dendrobates pumilio* from Bocas del Toro, Panama. J Chem Ecol.

[20] Saporito RA, Donnelly MA, Madden AA, Garraffo HM, Spande TF (2010). Sex-related differences in alkaloid chemical defenses of the dendrobatid frog *Oophaga pumilio* from Cayo Nancy, Bocas del Toro, Panama. J Nat Prod.

[21] Daly JW, Wilham JM, Spande TF, Garraffo HM, Gil R, Silva G (2007). Alkaloids in bufonid toads (*Melanophryniscus*): temporal and geographic determinants for two Argentinian species. J Chem Ecol.

[22] Daly JW, Garraffo HM, Spande TF, Yeh HJC, Peltzer PM, Cacivio PM (2008). Indolizidine 239Q and quinolizidine 275I. Major alkaloids in two Argentinian bufonid toads (*Melanophryniscus*). Toxicon.

[23] Garraffo HM, Andriamaharavo NR, Vaira M, Quiroga MF, Heit C, Spande TF (2012). Alkaloids from single skins of the Argentinian toad *Melanophryniscus rubriventris* (Anura, Bufonidae): an unexpected variability in alkaloid profiles and a profusion of new structures. Springerplus.

[24] Grant T, Colombo P, Verrastro L, Saporito RA (2012). The occurrence of defensive alkaloids in non-integumentary tissues of the Brazilian red-belly toad *Melanophryniscus simplex* (Bufonidae). Chemoecology.

[25] Shine R (1979). Sexual selection and sexual dimorphism in the Amphibia. Copeia.

[26] Monnet JM, Cherry MI (2002). Sexual size dimorphism in anurans. Proc R Soc London Ser B Biol Sci.

[27] Myers CW, Daly JW, Malkin B (1978). A dangerously toxic new frog (*Phyllobates*) used by Emberá indians of western Colombia, with discussion of blowgun fabrication and dart poisoning. Bull Am Museum Nat Hist.

[28] Daly JW, Kaneko T, Wilham JM, Garraffo HM, Spande TF, Espinosa A (2002). Bioactive alkaloids of frog skin: combinatorial bioprospecting reveals that pumiliotoxins have an arthropod source. Proc Natl Acad Sci U S A.

[29] Stynoski JL, Torres-Mendoza Y, Sasa-Marin M, Saporito RA (2014). Evidence of maternal provisioning of alkaloid-based chemical defenses in the strawberry poison frog *Oophaga pumilio*. Ecology.

[30] Santos RR, Grant T (2011). Diel pattern of migration in a poisonous toad from Brazil and the evolution of chemical defenses in diurnal amphibians. Evol Ecol.

[31] Santos RR, Leonardi SB, Caorsi VZ, Grant T (2010). Directional orientation of migration in an aseasonal explosive-breeding toad from Brazil. J Trop Ecol.

[32] Caorsi VZ, Colombo P, Freire MD, Amaral IB, Zank C, Borges-Martins M (1973). Natural history, coloration pattern and conservation status of the threatened south Brazilian red bellied toad, *Melanophryniscus macrogranulosus* Braun. Herpetol Notes.

[33] Nishida R (2002). Sequestration of defensive substances from plants by Lepidoptera. Annu Rev Entomol.

[34] Donnelly MA (1991). Feeding patterns of the strawberry poison frog, *Dendrobates pumilio* (Anura: Dendrobatidae). Copeia.

[35] Daly JW, Myers CW, Warnick JE, Albuquerque EX (1980). Levels of batrachotoxin and lack of sensitivity to its action in poison-dart frogs (*Phyllobates*). Science.

[36] Daly JW, Garraffo HM, Spande TF, Jaramillo CA, Rand AS (1994). Dietary source for skin alkaloids of poison frogs (Dendrobatidae)?. J Chem Ecol.

[37] Saporito RA, Isola M, Maccachero VC, Condon K, Donnelly MA (2010). Ontogenetic scaling of poison glands in a dendrobatid poison frog. J Zool.

[38] Barnett CA, Bateson M, Rowe C, Barnett CA (2014). Better the devil you know: avian predators find variation in prey toxicity aversive. Biol Lett.

[39] Mina AE, Ponti AK, Woodcraft NL, Johnson EE, Saporito RA (2015). Variation in alkaloid-based microbial defenses of the dendrobatid poison frog *Oophaga pumilio*. Chemoecology.

[40] Halliday TR, Verrell PA (1988). Body size and age in amphibians and reptiles. J Herpetol.

[41] Wake DB, Castanet J (1995). A skeletochronological study of growth and age in relation to adult size in *Batrachoseps attenuatus*. J Herpetol.

[42] Ento K, Matsui M (2002). Estimation of age structure by skeletochronology of a population of *Hynobius nebulosus* in a breeding season (Amphibia, Urodela). Zoolog Sci.

[43] Morrison C, Hero JM, Browning J (2004). Altitudinal variation in the age at maturity, longevity, and reproductive lifespan of anurans in subtropical Queensland. Herpetologica.

[44] Yamamoto T, Ota H, Chiba S (2011). The age structure of a breeding population of *Hynobius lichenatus* (Amphibia, Caudata). Curr Herpetol.

[45] Sinsch U (2015). Review: skeletochronological assessment of demographic life-history traits in amphibians. Herpetol J.

[46] Rozenblut B, Ogielska M (2005). Development and growth of long bones in European water frogs (Amphibia: Anura: Ranidae), with remarks on age determination. J Morphol.

[47] Bokermann WCA (1967). Observações sobre *Melanophryniscus moreirae* (Mir. Rib.) (Amphibia—Brachycephalidae). An Acad Bras Cienc.

[48] Marques RM, Colas-Rosas PF, Toledo LF, Haddad CFB (2006). Amphibia, Anura, Bufonidae, *Melanophryniscus moreirae*: distribution extension. Check List.

[49] Weber LN, Procaci LDS, Salles R, Silva SP, Corrêa AL, Carvalho-e-Silva SP (2007). Amphibia, Anura, Bufonidae, *Melanophryniscus moreirae*: distribution extension. Check List.

[50] Carvajalino-Fernández JM, Jeckel AM, Indicatti RP (2013). *Melanophryniscus moreirae* (Amphibia, Anura, Bufonidae): dormancy and hibernacula use during cold season. Herpetol Bras.

[51] Kusrini MD, Alford RA (2006). The application of skelectochronology to estimate ages of three species of frogs in West Java, Indonesia. Herpetol Rev.

[52] Rasband WS (2014). ImageJ, version 1.48.

[53] Hemelaar A (1985). An improved method to estimate the number of year rings resorbed in phalanges of *Bufo bufo* (L.) and its application to populations from different latitudes and altitudes. Amphibia-Reptilia.

[54] Castanet J, Smirina EM (1990). Introduction to the skeletochronological method in amphibians and reptiles. Ann Sci Nat Zool.

[55] Sagor ES, Ouellet M, Barten E, Green DM (1998). Skeletochronology and geographic variation in age structure in the wood frog, *Rana sylvatica*. J Herpetol.

[56] Guarino FM, Lunardi S, Carlomagno M, Mazzotti S (2003). A skeletochronological study of growth, longevity, and age at sexual maturity in a population of *Rana latastei* (Amphibia, Anura). J Biosci.

[57] Piantoni C, Ibargüengoytía N, Cussac V (2006). Age and growth of the Patagonian lizard *Phymaturus patagonicus*. Amphibia-Reptilia.

[58] Daly JW, Spande TF, Garraffo HM (2005). Alkaloids from amphibian skin: a tabulation of over eight-hundred compounds. J Nat Prod.

[59] Rice WR (1989). Analyzing tables of statistical tests. Evolution.

[60] Paradis E, Claude J, Strimmer K (2004). APE: analyses of phylogenetics and evolution in R language. Bioinformatics.

[61] R Core Team (2015). R: a language and environment for statistical computing.

