# Supplementary material for: The relationship between poison frog chemical defenses and age, body size, and sex
Source: Front Zool. 2015 Oct 1;12:27. doi: 10.1186/s12983-015-0120-2 (PMC4591705; doi:10.1186/s12983-015-0120-2)
Supplement: Additional file 1: — Results of t -tests comparing the sizes (snout–vent length) of adult females and males of the same age. (PDF 34 kb) [file 12983_2015_120_MOESM1_ESM.pdf]

Additional file 1

Results of *t*-tests comparing the sizes (snout–vent length) of adult females and males of the same age. All *P*-values remained significant following sequential Bonferroni test for table-wide significance.

Age (years)	Mean female size (mm)	Mean male size (mm)	*t*	DF	*P*
3	26.32	23.76	6.1489	2	**0.02544**
4	26.52	23.28	7.3265	19	**6.032e-07**
5	26.03	23.52	5.0034	17	**0.0001088**
6	26.068	23.400	3.3004	5	**0.02147**
