# Supplementary material for: The relationship between poison frog chemical defenses and age, body size, and sex
Source: Front Zool. 2015 Oct 1;12:27. doi: 10.1186/s12983-015-0120-2 (PMC4591705; doi:10.1186/s12983-015-0120-2)
Supplement: Additional file 2: — Defensive chemicals ranked by quantity in individual skins of Melanophryniscus moreirae juveniles. (PDF 69 kb) [file 12983_2015_120_MOESM2_ESM.pdf]

Additional file 2. Defensive chemicals ranked by quantity in individual skins of *Melanophryniscus moreirae* juveniles.

	Rank Order by Quantity (µg per individual)													
**MZUSP**	**1**	**2**	**3**	**4**	**5**	**6**	**7**	**8**	**9**	**10**	**11**	**12**	**13**	**Color Code**
**154107**	**257**	**27**	**22**	**16**	**15**	**10**	**4**	**2**	**2**	**2**				**Bufotenine**
**154108**	**195**	**121**	**35**	**11**	**4**	**4**	**4**	**3**	**3**	**3**	**1**	**1**	**0**	5,6,8-I **277E**
**154147**	**2**													5,6,8-I **279F**
**154148**	**62**													5,6,8-I **279F** iso 1
**154149**	**20**	**5**	**2**											5,6,8-I **295G**
**154150**	**34**	**22**	**9**	**4**	**2**	**1**	**1**	**1**						5,8-I **241K**
**154151**	**7**													5,8-I **297G**
														aPTX **323B**
														aPTX **323B** iso 1
														aPTX **337D**
														hPTX **281K**
														PTX **265D**
														PTX **267C**
														PTX **267C** iso 1
														PTX **295F**
														PTX **323A**
														UNCLASS **237W**
														TRI **265S**
														UNCLASS **251GG**

Chemicals are color-coded and their quantities (µg per individual) are reported in each cell. Abbreviations: 5,6,8-I, 5,6,8-trisubstituted indolizidine; 5,8-I, 5,8-disubstituted indolizidine; aPTX, allopumiliotoxin; hPTX, homopumiliotoxin; iso, isomer; PTX, pumiliotoxin; TRI, tricyclic;Unclass, unclassified as to structure.
