# Supplementary material for: The relationship between poison frog chemical defenses and age, body size, and sex
Source: Front Zool. 2015 Oct 1;12:27. doi: 10.1186/s12983-015-0120-2 (PMC4591705; doi:10.1186/s12983-015-0120-2)
Supplement: Additional file 3: — Additional details on age calculation methods. (PDF 140 kb) [file 12983_2015_120_MOESM3_ESM.pdf]

Additional file 3
Methods of age calculation

We estimated the number of missing lines of arrested growth (LAGs) by calculating the difference between the perimeter of the resorption line (RL) and the mean perimeter of each of the visible LAGs of the smallest individuals. If the difference exceeded 1 SD we added the corresponding number of inferred LAGs to the number of observed LAGs for age calculation; if the difference was < 1 SD, evidence of LAG destruction is lacking and we calculated age based only on observed LAGs.

Several other methods have been proposed to estimate the number of LAGs destroyed by endosteal resorption, including those of Sagor et al. (1998), Guarino et al. (2003), and Piantoni et al. (2006).

Sagor et al. (1998). This method begins by plotting the measurements of the first and second innermost visible LAGs. If the difference between the innermost visible LAG diameter for a given individual is > 2 SD greater than the group mean, LAG resorption is inferred to have occurred and the first visible LAG is considered LAG 2. Sagor et al. (1998) also treated the periosteal outer margin as a LAG because the frogs they studied were captured shortly after emergence from hibernation, which was not the case in the present study.

Guarino et al. (2003). In this method, the RL perimeter of each adult is compared to the perimeter of the first visible LAGs. If the RL perimeter exceeds the perimeter of the first visible LAG in other individuals, one inferred LAG is added to the number of visible LAGs. Guarino et al. (2003) also compared the perimeter of the first visible LAG with the periosteal outer margin of juveniles close to first overwintering, but this was not applicable in our study because all collected juveniles already exhibited well-defined LAGs.

Piantoni et al. (2006). These authors used the software SigmaPlot version 13 (Systat Software, Inc) to perform a quadratic regression of RLs and LAGs on SVL. The number of resorbed LAGs of an individual of a given SVL is calculated by observing the RL perimeter estimated for that SVL and determining the number of LAGs of the same perimeter that are present in smaller individuals, which is equal to the number of LAG regression curves intersected by a horizontal line drawn from the RL regression curve at that SVL; if the RL of a given individual does not exceed the estimated LAG perimeters for that SVL, loss of LAGs is assumed not to have occurred.

To determine the sensitivity of our results to choice of age calculation method, we repeated all analyses using each of these methods as well. The resulting age structure estimates are summarized in Appendix S1 Table 1. Despite the variation in age estimates, the results of the multiple regression analyses remain unchanged (Appendix S1 Tables 2–4).

Additional file 3 Table 1. *Melanophryniscus moreirae* population age structure using different age estimation methods (see main text for references). AM: age at maturity, age of the youngest adults; PRLS: potential reproductive lifespan; size: snout-vent length.

**This paper**							
Sex	Median Age (years)	Median Age (years)	Modal Age (years)	AM (years)	Mean Size at AM ± SE (mm)	Longevity (years)	PRLS (years)
Male	4.2 ± 0.2	4	4	2	21.7 ± 0.9	6	4
Female	4.9 ± 0.3	5	5 and 6	3	26.3 ± 0.0	6	3
**Sagor et al. 1998**							
Sex	Mean Age ± SE (years)	Median Age (years)	Modal Age (years)	AM (years)	Mean Size at AM ± SE (mm)	Longevity (years)	PRLS (years)
Male	5.4 ±0.2	5	5	4	23.4 ± 0.3	8	4
Female	6.0 ± 0.2	6	5 and 7	5	26.3 ± 0.4	7	2
**Guarino et al. 2003**							
Sex	Mean Age ± SE (years)	Median Age (years)	Modal Age (years)	AM (years)	Mean Size at AM ± SE (mm)	Longevity (years)	PRLS (years)
Male	5.3 ±0.1	5	5	4	23.2 ± 0.5	7	3
Female	6.0 ± 0.2	6	6	5	26.6 ± 0.5	7	2
**Piantoni et al. 2006**							
Sex	Mean Age ± SE (years)	Median Age (years)	Modal Age (years)	AM (years)	Mean Size at AM ± SE (mm)	Longevity (years)	PRLS (years)
Male	4.4 ± 0.1	4	4	3	23.8 ± 0.2	7	4
Female	4.6 ± 0.3	5	5	4	26.5 ± 0.5	6	2

Additional file 3 Table 2. Results of non-parametric multiple regression analyses (9999 permutations; two-tailed tests; 3 and 58 degrees of freedom) of Brazilian red-belly toad chemical defenses in relation to sex, skin mass, and age estimated following the method of Sagor et al. (1998). Statistically significant *P*-values are marked in **bold**.

	Alkaloid Richness			Alkaloid Quantity			Bufotenine Quantity		
	Sex	Skin Mass	Age	Sex	Skin Mass	Age	Sex	Skin Mass	Age
Regression coefficient	-1.066768	0.013099	1.089503	108.0503	1.5887	42.5084	15.61609	0.58844	5.38879
P	0.2707	0.1082	**0.0151**	0.0770	**0.0030**	0.1242	0.4008	**0.0004**	0.5069
R^2		0.2754126			0.3677446			0.3431981	
R^2-adj		0.2379339			0.3350418			0.3092256	
F		7.348518			11.24503			10.10223	
P		**0.0003**			**0.0001**			**0.0001**	

Additional file 3 Table 3. Results of non-parametric multiple regression analyses (9999 permutations; two-tailed tests; 3 and 58 degrees of freedom) of Brazilian red-belly toad chemical defenses in relation to sex, skin mass, and age estimated using the method of Guarino et al. (2003). Statistically significant *P*-values are marked in **bold**.

	Alkaloid Richness			Alkaloid Quantity			Bufotenine Quantity		
	Sex	Skin Mass	Age	Sex	Skin Mass	Age	Sex	Skin Mass	Age
Regression coefficient	-<br>0.5776610	0.0065844	1.2351079	129.4493	1.2945	50.865<br>7	15.69639	0.59671	3.40596
P	0.5680	0.4898	**0.0111**	**0.0428**	**0.0355**	0.0917	0.4307	**0.0025**	**0.**7116
R^2		0.2802453			0.372606			0.3399654	
R^2-adj		0.2430166			0.3401546			0.3058257	
F		7.527669						9.958061	
P		**0.0004**			**0.0001**			**0.0001**	

Additional file 3 Table 4. Results of non-parametric multiple regression analyses (9999 permutations; two-tailed tests; 3 and 58 degrees of freedom) of Brazilian red-belly toad chemical defenses in relation to sex, skin mass, and age estimated using the method of Piantoni et al. (2006). Statistically significant *P*-values are marked in **bold**.

	Alkaloid Richness			Alkaloid Quantity			Bufotenine Quantity		
	Sex	Skin Mass	Age	Sex	Skin Mass	Age	Sex	Skin Mass	Age
Regression coefficient	-0.7808353	0.0079642	1.5219014	107.297	1.624	38.438	13.55504	0.63179	1.41681
P	0.4164	0.3343	**0.0015**	0.0868	**0.0025**	0.2040	0.4761	**0.0003**	0.8789
R^2		0.3261683			0.3593323			0.3386551	
R^2-adj		0.2913149			0.3261943			0.3044476	
F		9.3583			10.84352			9.900025	
P		**0.0001**			**0.0001**			**0.0003**	
