# Supplementary material for: The relationship between poison frog chemical defenses and age, body size, and sex
Source: Front Zool. 2015 Oct 1;12:27. doi: 10.1186/s12983-015-0120-2 (PMC4591705; doi:10.1186/s12983-015-0120-2)
Supplement: Additional file 4: — Results of non-parametric multiple regression analyses of Brazilian red-belly toad chemical defenses in relation to sex, skin mass, and age following removal of juveniles. (PDF 51 kb) [file 12983_2015_120_MOESM4_ESM.pdf]

Table S1. Results of non-parametric multiple regression analyses (9999 permutations; two-tailed tests; 3 and 51 degrees of freedom) of Brazilian red-belly toad chemical defenses in relation to sex, skin mass, and age following exclusion of the seven juvenile specimens. Statistically significant *P*-values are marked in **bold**.

	Alkaloid Richness			Alkaloid Quantity			Bufotenine Quantity		
	Sex	Skin Mass	Age	Sex	Skin Mass	Age	Sex	Skin Mass	Age
Regression coefficient	0.2207798	-<br>0.0037913	1.1439251	149.7665<br>1	1.24970	48.684<br>8	24.74898<br>6	0.570354	-<br>0.067456
P	0.8909	0.7964	**0.0205**	0.1583	0.1787	0.1169	0.4527	**0.0483**	0.9945
R^2	0.1100187			0.3212327			0.2443528		
R^2-adj	0.05766691			0.2813052			0.199903		
F	7.348518			8.045402			5.497271		
P	0.1095			**0.0001**			**0.0025**		
